# Primary Squamous Cell Carcinoma of the Cecum Presenting as Spontaneous Perforation

**DOI:** 10.7759/cureus.10510

**Published:** 2020-09-17

**Authors:** Luisa M Recinos, Sonmoon Mohapatra, Arkady Broder, Imran Saeed

**Affiliations:** 1 Internal Medicine, Saint Peter’s University Hospital – Rutgers Robert Wood Johnson School of Medicine, New Brunswick, USA; 2 Gastroenterology and Hepatology, Saint Peter’s University Hospital – Rutgers Robert Wood Johnson School of Medicine, New Brunswick, USA; 3 Surgery, Saint Peter’s University Hospital – Rutgers Robert Wood Johnson School of Medicine, New Brunswick, USA

**Keywords:** squamous cells carcinoma, perforated colon, colon cancer and colon polyps, squamous cell neoplasm, perforated, colorectal neoplasia, colorectal cancer, lower gi or colorectal surgery

## Abstract

Squamous cell carcinoma (SCC) of the colon is an extremely rare condition, and its pathogenesis is not fully understood. Bowel perforation is a very infrequent manifestation of colonic SCC, and only a few cases have been reported in the literature involving sigmoid and splenic flexure perforation. To the best of our knowledge, no cases of ileocecal perforation have been documented in the literature. Here we present a case of cecal SCC that presented with bowel perforation, necessitating emergent surgical intervention. Histopathological examination showed SCC with lymph node metastasis. This case reviews current knowledge and highlights the rare manifestation that these rare tumors can present.

## Introduction

Primary squamous cell carcinoma (SCC) of the colon is an extremely rare entity accounting for only 0.1-0.25/1000 of all colorectal malignancies [1]. Its pathogenesis is not fully understood, but it has been associated with inflammation, infectious processes, exposure to radiation, and squamous differentiation of existing colonic adenomas. It usually presents in the fifth to sixth decades of life and most commonly in women [2].

The clinical presentation of colonic SCC is described to be similar to that of colorectal adenocarcinoma; however, bowel perforation is an unusual presenting manifestation. To establish the diagnosis of primary colonic SCC, other primary tumors must be excluded as part of the diagnostic criteria. Diagnostic criteria also include histological confirmation and lack of continuity of the tumor with anal epithelium or squamous lined fistula [3].

Currently, there are no treatment guidelines for this condition. Surgical resection has been commonly used with the aim of complete excision with negative margins. More recently, in addition to surgery, chemotherapy has been used for the treatment of this condition. SCC of the colon has been reported to have higher mortality rates than colonic adenocarcinoma [1]. Here we present a case of SCC of the cecum in a patient who presented with bowel perforation requiring emergent surgical intervention.

## Case presentation

A 61-year-old African American female presented to the emergency department with a two-day history of worsening right lower quadrant pain and melena. She also reported 20 lbs of unintentional weight loss and intermittent fever. She had no personal or family history of malignancy. 

**Figure 1:**
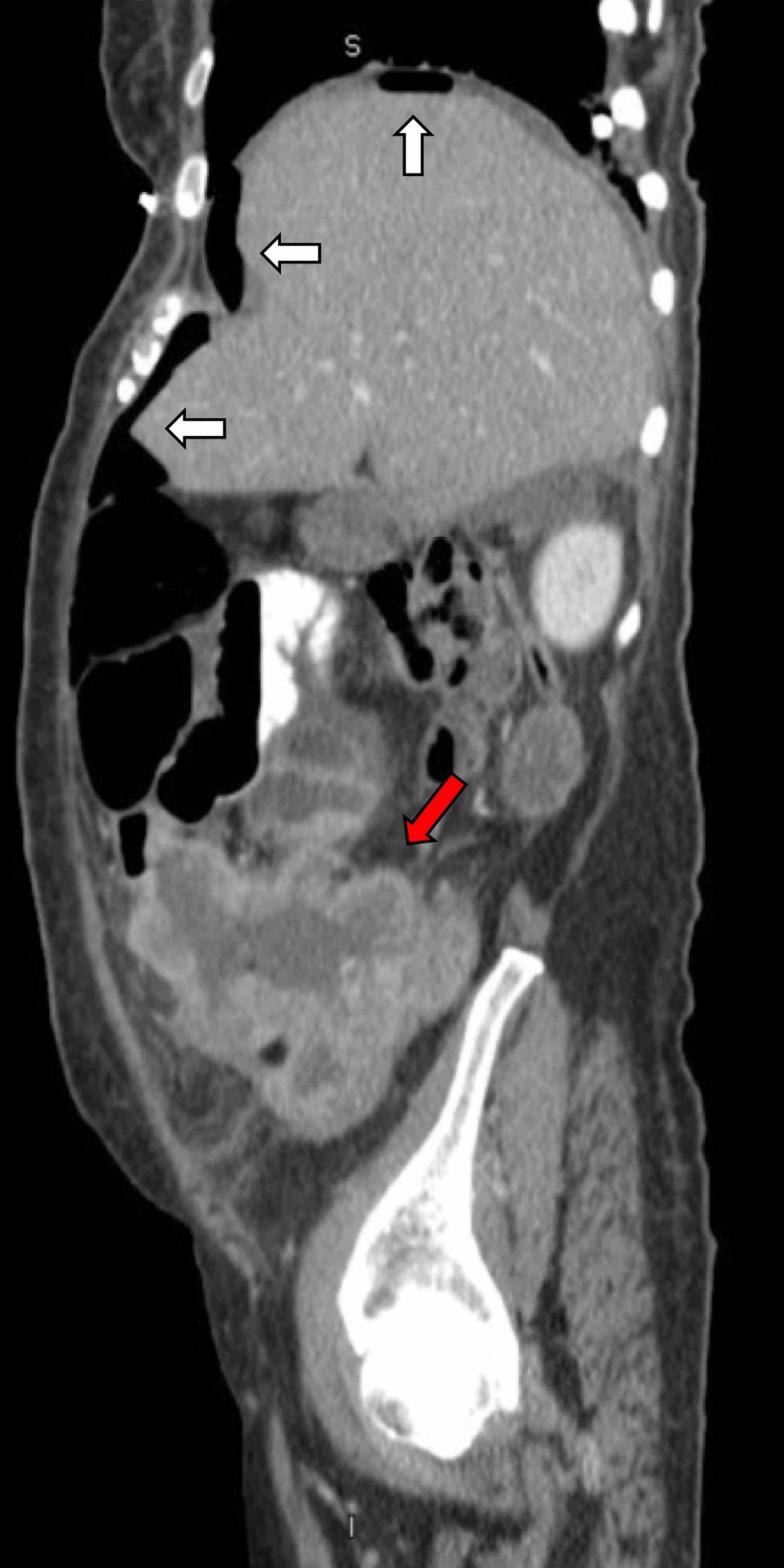
CT scan of the abdomen Pneumoperitoneum is shown by white arrows; irregular bowel thickening was noted in right lower quadrant (red arrow).

On physical examination, her abdomen was distended and diffusely tender to palpation. A CT scan of the abdomen and pelvis showed intraperitoneal free air (Figure 1) and ascites, suggestive of a ruptured hollow viscus. Low-grade small bowel obstruction was noted with a transition point at a site of marked irregular bowel thickening in the right lower quadrant (Figure 1). An emergent exploratory laparotomy was performed. Fecal peritonitis and a perforated necrotic ileocecal mass were found. The mass was invading two small bowel loops, and no direct extension was noted to the adnexa. En bloc removal of that mass was performed along with right hemicolectomy and the creation of an end ileostomy. A separate segment of the small bowel loop that was invading the tumor was also resected.

**Figure 2:**
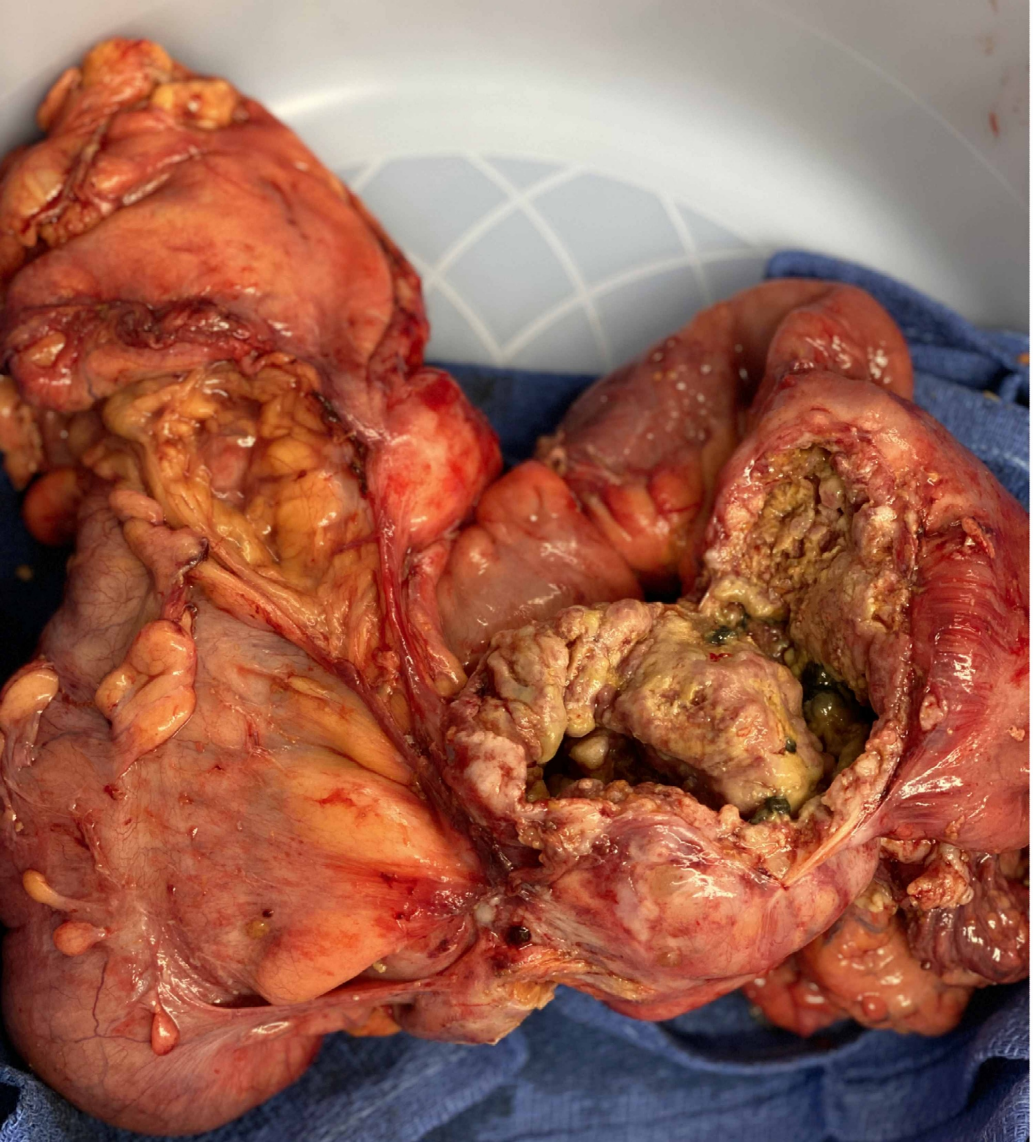
Operative specimen Resected mass measured 8 cm x 6 cm x 4 cm.

**Figure 3:**
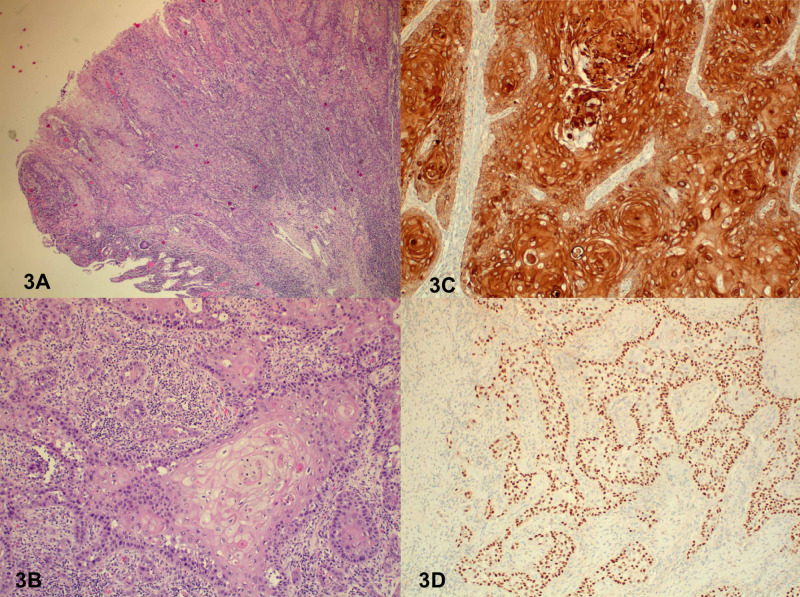
Microscopic evaluation A) Moderately differentiated squamous cell carcinoma (grade 2) and small bowel. (B) High power field. (C) Positive Ck5/6 immunostain. (D) Positive P63 immunostain.

The pathological gross description showed two segments of intestine adherent to one another by a perforated mass. The mass was white-gray, necrotic, firm, and measured 8 cm x 6 cm x 4 cm (Figure 2). Microscopic evaluation revealed a moderately differentiated SCC (grade 2) (Figures 3A, 3B). The mesenteric tissues showed multiple lymph nodes; 2 out of 17 lymph nodes were positive for metastatic carcinoma. Immunostains showed Ck5/6 (+) (Figure 3C) and P63 (+) (Figure 3D). The pathological stage was pT4bN1b. Additional gynecological and pulmonary evaluations did not reveal any concern for a primary malignancy.

## Discussion

SCC of the gastrointestinal tract is common in the esophagus and anus; however, it rarely affects the colon and rectum. The first case of SCC of the colon was described in 1919 [2]. Since then, 150 cases of colorectal SCC have been reported, and 65 cases were found in the English literature [1]. The most common location is the rectum followed by the right colon, with the majority of cases presenting in an advanced stage. The etiology of the SCC is not clearly known. It has been proposed to be associated with chronic inflammation, as seen with ulcerative colitis [4]. Suggested infectious etiologies include HPV (human papillomavirus), schistosomiasis, and *Entamoeba histolytica*. A history of previous surgical procedures or radiation therapy has been found in patients with colonic SCC [5]. Hicks and Cowling described the possibility of pluripotent stem cells capable of squamous differentiation [6]. Our patient didn’t have any of the apparent risk factors for primary SCC of the colon, raising the concern that other underlying unknown predisposing factors may play a role in this condition.

The clinical manifestation of primary SCC of the colon has been described to be comparable to colorectal adenocarcinoma. Frequently reported symptoms include rectal bleeding, abdominal pain, change in bowel habits, weight loss, and bowel obstruction [2]. In the literature review, we only found three cases describing sigmoid or splenic flexure perforation, but none with cecal perforation [7-9]. Given the unclear underlying mechanism of these malignancies and how infrequently they present, the following criteria have been proposed by Williams et al. to diagnose SCC of the colon: (1) no evidence of a primary SCC elsewhere that could be a source of metastatic or direct extension to the bowel, (2) the affected segment of the bowel is not in continuity with a squamous lined fistula, (3) there is no continuity between the tumor and the anal squamous epithelium, and (4) confirmation of SCC by histological analysis [3]. Our patient fulfilled these criteria. Hypercalcemia and hyperleukocytosis have been the reported biochemical abnormalities in some cases; the latter was evident in our patient case [9].

The rarity of these tumors and the lack of randomized controlled trials impede the creation of treatment guidelines. Generally, surgical intervention has been routinely performed with the more recent use of neoadjuvant chemotherapy (5-fluorouracil and mitomycin C) and radiation therapy. The overall five-year survival rate for these patients is 30%-35%, which is less than those with adenocarcinoma of the colon [1, 10]. If available, serum SCC antigen levels can be used to monitor recurrence [11]. It has been suggested that six-monthly colonoscopies for two years should be performed [12].

## Conclusions

Primary SCC of the colon is a rare entity, and its pathogenesis is not fully understood. Given the small number of cases reported, there are no established treatment protocols for this condition. Surgical intervention has been routinely employed in addition to adjuvant chemoradiation and followed by close endoscopic surveillance. Documentation of this case is crucial as it presented in an unusual manner with bowel perforation. We hope that our experience can help further understand this condition and help in the creation of clinical guidelines in the future.
